# Hemorrhagic Cholecystitis in a Patient with Cirrhosis and Rectal Cancer

**DOI:** 10.7759/cureus.7882

**Published:** 2020-04-29

**Authors:** Rony Shah, Linda C Klumpp, James Craig, Parth Patel, Jeffrey Jordan

**Affiliations:** 1 Internal Medicine, Citrus Memorial Hospital, Inverness, USA; 2 Surgery, Citrus Memorial Hospital, Inverness, USA

**Keywords:** cholecystitis, hemorrhagic cholecystitis, hemoperitoneum, cirrhosis, rectal cancer

## Abstract

Hemorrhagic cholecystitis is a rare presentation of acute calculous cholecystitis which presents with abdominal pain, jaundice, and gastrointestinal bleeding. It is a challenging diagnosis to make because it present similar to other common disorders such as calculous cholecystitis. We present a unique case of hemorrhagic cholecystitis in a patient with cirrhosis and rectal cancer.

A 66-year-old male with a history of rectal cancer, alcohol-induced cirrhosis, esophageal varices, stroke, paroxysmal atrial fibrillation, and hypertension presented to the emergency department with complaints of abdominal pain. Patient’s computed tomography (CT) scan revealed bleeding from the gallbladder with hemoperitoneum and thickening of the ascending colon. The patient underwent emergent surgery for hemorrhagic cholecystitis.

Hemorrhagic cholecystitis is associated with risk factors, including trauma, malignancy, renal failure, cirrhosis, and anticoagulation therapy. Imaging is not always reliable, but ultrasound and CT scan are the preferred options. Treatment options are surgical or nonsurgical approach depending on patient’s hemodynamic stability.

## Introduction

Hemorrhagic cholecystitis is a life-threatening complication of acute calculous cholecystitis. A hemorrhage within the gallbladder can occur for a variety of reasons, such as obstructive cholecystitis, biliary neoplasm, biliary parasites, bleeding disorder, percutaneous intervention, and trauma [1]. It is a challenging diagnosis to make because its presentation is similar to calculous cholecystitis. Hemorrhagic cholecystitis typically presents with abdominal pain, jaundice, and gastrointestinal bleeding which is found in 22% of cases [2]. We present a unique case of hemorrhagic cholecystitis in a patient with cirrhosis and rectal cancer. 

## Case presentation

**Figure 1:**
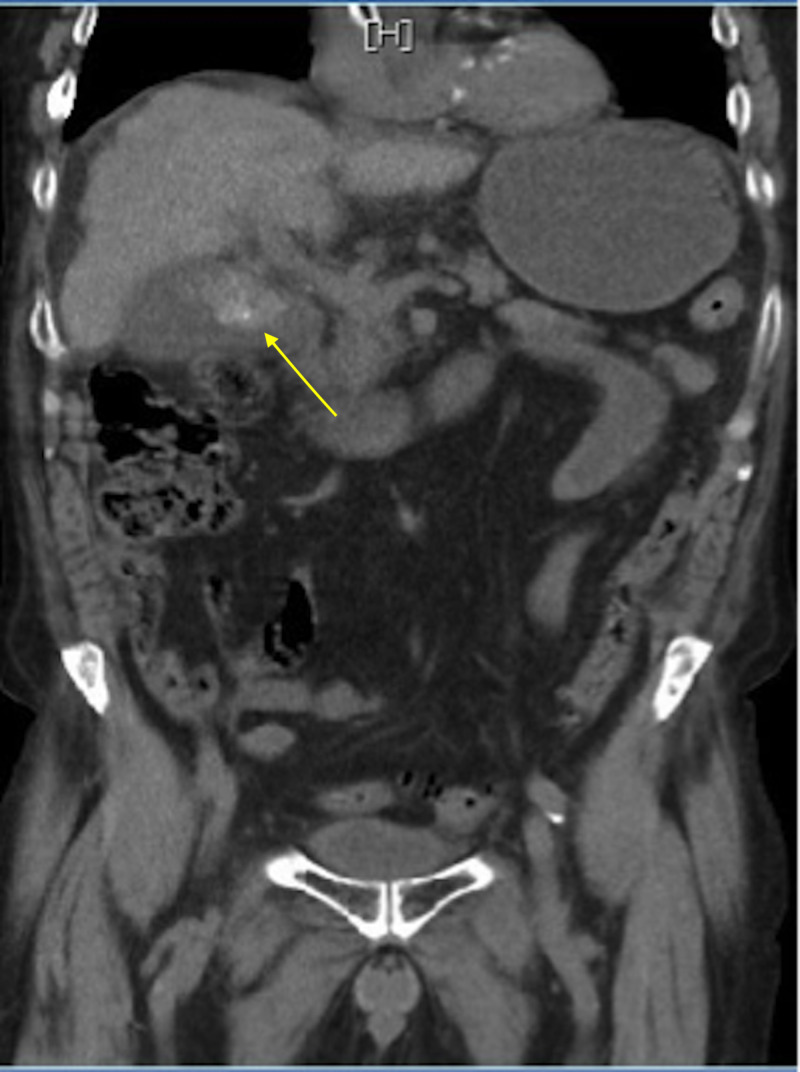
CT of the abdomen and pelvis without contrast Cholelithiasis with enlarged gallbladder or liver mass

A 66-year-old male presented to the emergency department (ED) with complaints of abdominal pain. The patient provided a past medical history of rectal cancer, alcohol-induced cirrhosis, esophageal varices, stroke, paroxysmal atrial fibrillation, and hypertension. Vital signs at admission were temperature 99 degree Fahrenheit, heart rate 111 beats per minute, respiratory rate 19 blood breaths per minute, pressure 132/82 mmHg, and oxygen saturation 95%. Physical examination was positive for right upper quadrant tenderness. Laboratory results showed white blood count 14,000/mm^3^, hemoglobin 9.5 g/dL, platelet count 77 x 10^9^/L, aspartate aminotransferase 19 U/L, alanine transaminase 12 U/L, alkaline phosphatase 85 U/L, prothrombin time/international normalized ratio 36/1.3. A CT of the abdomen and pelvis without contrast revealed cirrhotic liver, cholelithiasis, and a mass extending to the gallbladder from the medial segment of the left hepatic lobe (Figure 1).

**Figure 2:**
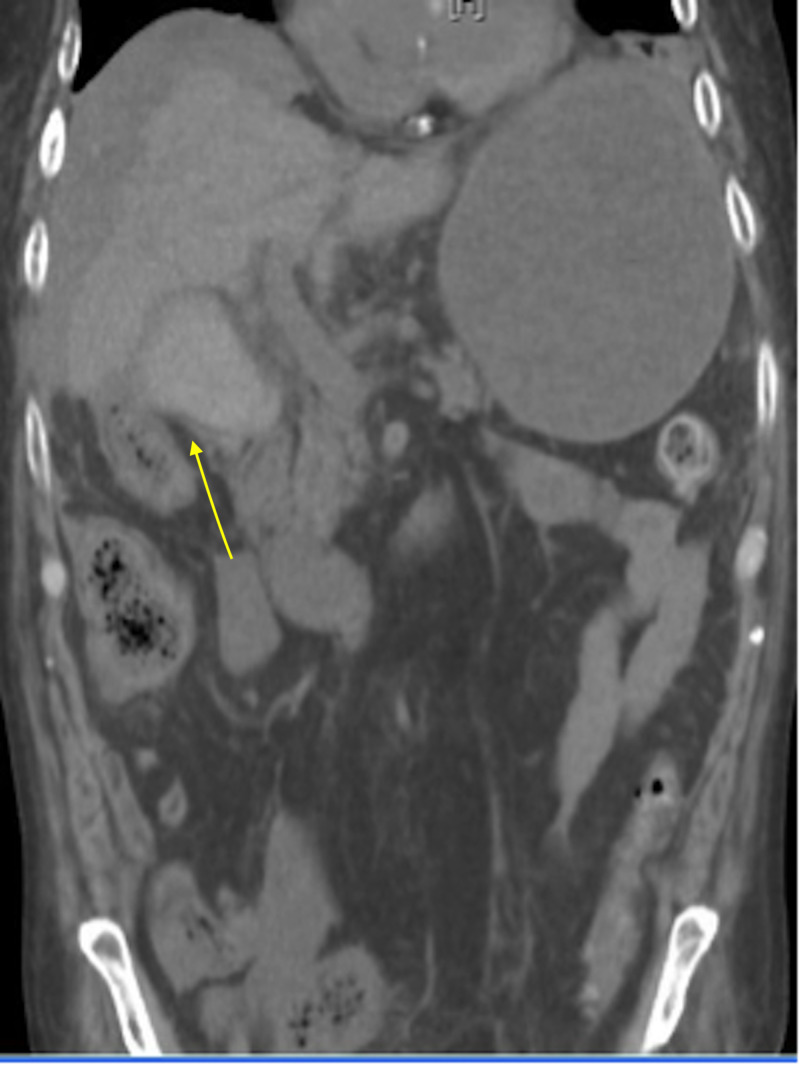
CT of the abdomen and pelvis without contrast Hemorrhage within the gallbladder resulting in a subcapsular hematoma

Ultrasound of the right upper quadrant revealed cholelithiasis with a distended gallbladder. The patient reported he had been recently treated with neoadjuvant chemotherapy and radiation for rectal cancer. His positron emission tomography/CT scan and MRI were negative one month prior to his ED visit. He developed multifocal atrial tachycardia and later wide QRS tachycardia soon after admission. The irregular rhythm was managed with beta-blocker therapy, oral metoprolol 50 mg twice a day. The patient had one episode of hematemesis and developed shock requiring aggressive intravenous fluid resuscitation and vasopressors. Rapid sequence intubation was required for acute hypoxemic respiratory failure due to possible aspiration. Bronchoscopy was performed afterwards and dark brownish secretions were suctioned. A repeat CT of the abdomen and pelvis without contrast showed bleeding from the gallbladder with hemoperitoneum and thickening of the ascending colon (Figure 2). The patient was emergently taken to surgery and underwent laparoscopic cholecystectomy with evacuation and drainage of intra-abdominal hematoma/hemoperitoneum and abscess. During surgery, a large hemoperitoneum was found throughout the abdomen, and after evacuating 1.5 L of cold blood and bile, the gallbladder was visualized. Bright red blood was oozing from the cystic artery, and the gallbladder was perforated with leakage of bile and blood throughout the abdomen. Active bleeding from the cystic artery was stopped with a staple because artery dissection and placement of a clip were not possible. The patient had two drains placed intra-abdominally, and his gallbladder was sent for pathology. He remained hemodynamically stable and was extubated four days later. Gallbladder pathology did not show any evidence of malignancy. Intra-abdominal cultures were positive for Klebsiella pneumoniae and Enterobacter cloacae due to gallbladder perforation. The patient was treated with appropriate antimicrobial therapy (pipercillin/tazobactam and meropenem) per culture sensitivity for seven days. He had an outpatient follow-up with the general surgeon in two weeks, and no complications were noted. 

## Discussion

In 1979, Shah and Clegg described hemobilia caused by cholecystitis as hemorrhagic cholecystitis [3,4]. Hemorrhagic cholecystitis can be difficult to diagnosis because it presents similarly to acute calculous cholecystitis and imaging is unreliable. Associated risk factors include trauma, malignancy, renal failure, cirrhosis, and anticoagulation therapy [5]. In our case, the patient had rectal cancer and history of cirrhosis. Cholelithiasis-related microbleeding of the gallbladder accounts for 9% of the hemobilia cases [3]. Hemorrhagic cholecystitis occurs due to transmural inflammation of the gallbladder wall leading to ischemia and mucosal breakdown, erosion into gallbladder vessels, and hemorrhage into the gallbladder lumen or abdominal cavity [6]. In majority of the reported cases, common presentation is right upper quadrant pain associated with fever and leukocytosis. Complications such as peritonitis, hematemesis, and melena may occur [6]. Hemorrhagic cholecystitis is associated with high mortality and morbidity, especially with gallbladder perforation and massive hemorrhage [7]. The mortality and morbidity rates associated with gallbladder perforation range between 15%-20% and 32%-58%, respectively [8]. Our patient presented with abdominal pain, leukocytosis, hematemesis, and gallbladder perforation leading to hemoperitoneum. Abdominal ultrasound is the investigation of choice for diagnosing upper-right quadrant pathology. Ultrasound findings typically show gallbladder wall thickening, intraluminal membranes, and nonshadowing, nonmobile intraluminal echogenic material [2,9]. A previous study by Chinn et al. revealed that 74% of patients reviewed had similar ultrasound findings [6]. CT findings typically show contrast extravasation during the arterial phase, high attenuation within the gallbladder lumen and fluid-fluid layering [2,10]. Image findings may be able to distinguish acute calculous cholecystitis from hemorrhagic cholecystitis. In our case, the repeat CT showed bleeding from the gallbladder with hemoperitoneum. Treatment option for hemorrhagic cholecystitis is either cholecystectomy or cholecystostomy, which is preferred in patients too hemodynamically unstable for surgery. Our patient underwent laparoscopic cholecystectomy with evacuation of intra-abdominal hemoperitoneum and abscess. Although he required intensive perioperative care, he was fortunate enough to make a complete recovery. Hemorrhagic cholecystitis is a rare complication that needs to be considered in high-risk patients.

## Conclusions

Hemorrhagic cholecystitis is associated with a high mortality and morbidity due to its life-threatening complications. Hemorrhagic cholecystitis can be difficult to diagnose; however, if clinically suspected, it warrants prompt medical investigation. Physician awareness with early identification and treatment can help prevent complications and death.
